# Association of geriatric nutritional risk index with total testosterone in elderly adults in the US: evidence from NHANES 2011-2016

**DOI:** 10.3389/fendo.2024.1457956

**Published:** 2024-12-16

**Authors:** Tanjian Li, Na Jiang, Xin Liang, Xinya Li, Yaqin Li, Yuting Huang, Yu Wang

**Affiliations:** ^1^ School of Nursing, Jinan University, Guangzhou, Guangdong, China; ^2^ School of Health, Binzhou Polytechnic, Binzhou, Shandong, China; ^3^ School of Nursing, Jinan University, The First Affiliated Hospital of Jinan University, The Community Health Service Center of Jinan University, Guangzhou, Guangdong, China

**Keywords:** serum total testosterone, Geriatric Nutritional Risk Index, older adults, NHANES database, men’s health

## Abstract

**Background and objectives:**

There is growing evidence that deficiencies in specific nutrients can impact testosterone levels in older men. However, research examining the predictive value of overall nutritional status on testosterone levels remains limited. The Geriatric Nutritional Risk Index (GNRI) is an effective tool for assessing the nutritional status of the elderly. Therefore, this study aimed to investigate the potential correlation between the GNRI and serum total testosterone (TT).

**Methods:**

A representative sample of U.S. males aged 60 years and older who participated in the National Health and Nutrition Examination Survey (NHANES) cycles from 2011 to 2016 was utilized for this cross-sectional study. The research included a total of 829 older adults. Tandem mass spectrometry and liquid chromatography were employed to quantify TT. To examine the association between GNRI and TT, restricted cubic splines (RCS) and weighted multivariate regression analyses were conducted. Subgroup analysis was performed to identify the variables influencing the positive association between GNRI and TT. Additionally, a sensitivity analysis was carried out to compare the weighted and unweighted data.

**Results:**

After adjusting for all other factors, a positive association was found between GNRI and TT. The beta coefficient was 5.59, with a 95% confidence interval of 2.16 to 9.01, and a p-value of 0.003. Compared to the lowest quartile of GNRI (Q1), the second quartile (Q2), third quartile (Q3), and fourth quartile (Q4) significantly increased the level of TT. The beta coefficients for Q2, Q3, and Q4 were 70.15 (p = 0.022), 104.40 (p < 0.001), and 84.83 (p < 0.001), respectively. In subgroup analyses, statistically significant associations were observed among participants who did not have diabetes, had hypertension, and had a BMI of 24.9 or less. According to the sensitivity analysis, unweighted data also found GNRI to be associated with TT (beta = 3.09, P = 0.031).

**Conclusion:**

A positive correlation was identified between the GNRI and TT in the elderly male population of the United States. Further prospective studies with larger sample sizes are needed to confirm the causal relationship between GNRI and TT.

## Introduction

Testosterone is a hormone synthesized by the testes and adrenal glands, and it has a vital function in male reproductive well-being. It is involved in several processes, including male genital development, reproduction and sexual behavior (1). The ageing process is accompanied by a progressive decrease in testosterone levels, which is linked to a reduction in both body fat and lean body mass (2, 3). A number of studies have demonstrated that low serum testosterone levels in men are associated with a number of adverse effects, including decreased mobility and sexual function (e.g., fatigue, erectile dysfunction) (4), as well as metabolic changes in the body, such as reduced bone mineral density content, which is associated with decreased levels of testosterone in men, increasing the risk of osteoporosis and falls (5). Moreover, decreased levels of testosterone (TT) have been linked to a heightened likelihood of death in elderly males (6). Consequently, it is imperative to identify effective strategies for managing TT levels in older men.

Malnutrition is a significant global health issue that has numerous detrimental effects on physical health and clinical outcomes (7). It is particularly prevalent among the elderly, primarily due to inadequate nutrient intake (8). Research has demonstrated that deficiencies in specific nutrients, such as zinc, magnesium, and vitamin D, as well as low consumption of polyphenols, can disrupt the hypothalamic-pituitary-gonadal (HPG) axis in men. This disruption can subsequently lead to reduced testosterone levels in the body (9). However, to date, no one has investigated the association between nutrition and testosterone levels from the perspective of overall nutritional status, particularly in the elderly population. The Geriatric Nutritional Risk Index (GNRI) is a straightforward nutritional assessment tool designed for older adults, which can predict the prognosis and mortality associated with various diseases, such as chronic obstructive pulmonary disease (10), hypertension (11), diabetes mellitus (12), and prostate cancer (13), etc. The GNRI combines several medical indicators, including current weight, height, and serum albumin levels, using simple formulas. Due to its ease of use in clinical analysis and its objective calculations based on existing data, the GNRI can address the limitations of relying on a limited number of indicators and subjective evaluations (14).

The National Health and Nutrition Examination Survey (NHANES) is a thorough survey conducted to gather data on various factors such as demographic information, socioeconomic status, diet and health, physiological measurements, laboratory tests, and other relevant details (15). This study will use the NHANES survey to determine the link between GNRI and testosterone levels in the senior population. This will enhance the ability to forecast and handle TT levels from a nutritional standpoint.

## Methods

### Database and survey populations

**Figure 1:**
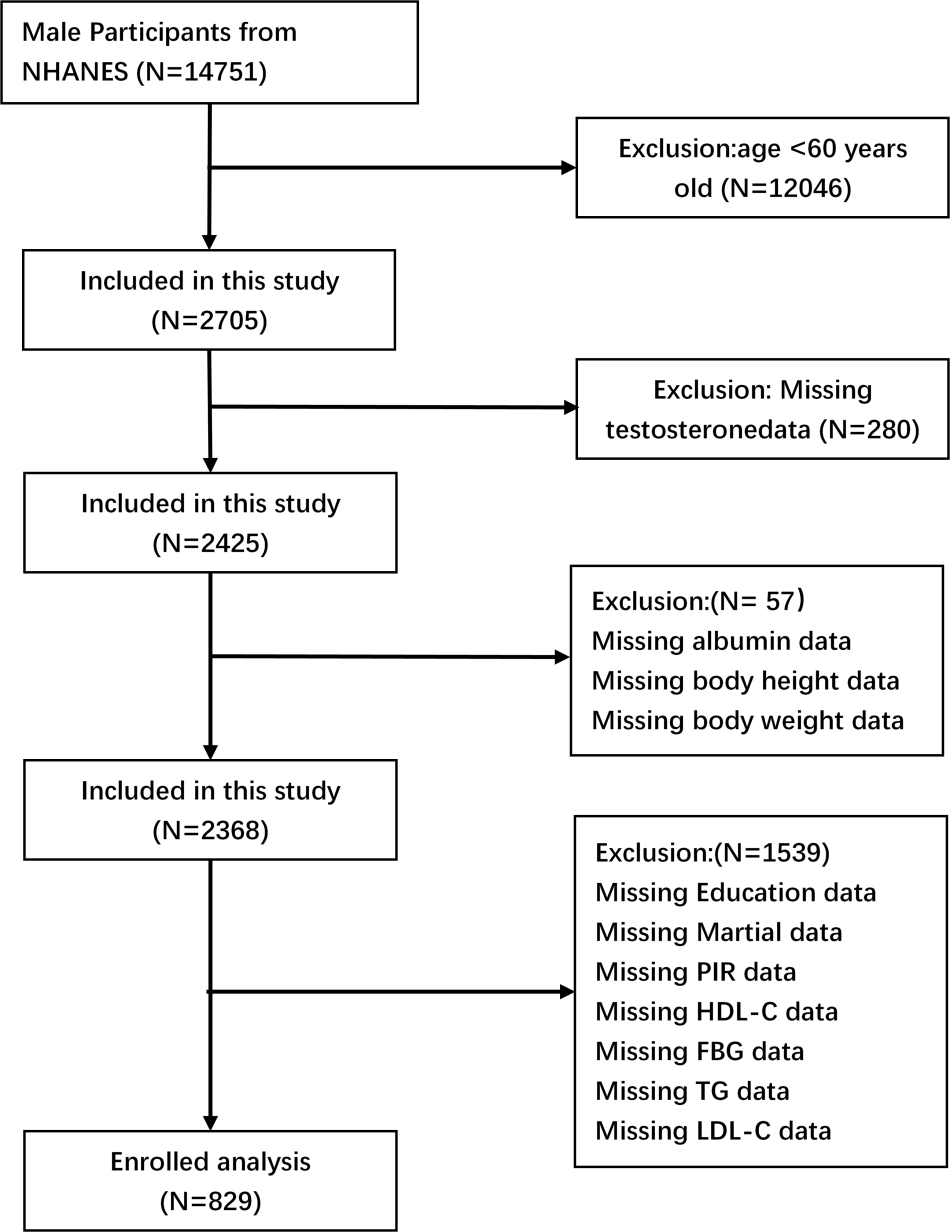
Flowchart depicting the screening procedure used to identify and select eligible individuals.

The data utilized in this investigation were obtained from the 2011-2012, 2013-2014, and 2015-2016 cycles of the National Health and Nutrition Examination Survey (NHANES). The Centers for Disease Control and Prevention (CDC) in the United States implemented NHANES using a multi-stage, complex stratified probability sampling methodology. This approach was designed to ensure the selection of a representative sample of adults and children from the U.S. population. The objective of the study was to assess the nutritional status of the participants. The study included a total of 14,751 male participants. The selected cycles were chosen because data on total testosterone levels are only available from 2011 to 2016. The study excluded 12,046 participants under the age of 60, 280 subjects who lacked total testosterone data, 57 GNRI subjects who were missing necessary data, and 1,539 subjects who lacked covariate data. The final analysis included 829 participants (**Figure 1**).

### Informed consent

NHANES is a dataset that is accessible to the public. Before starting any official inquiry, all participants must give both written and verbal agreement to take part in the research. The study has undergone evaluation and received approval from the ethical review committee of the National Centre for Health Statistics (NCHS). The NCHS IRB/ERB protocol number for 2011-2016 was #2011-17. The website (https://www.cdc.gov/nchs/nhanes/) provides access to all pertinent data.

### GNRI evaluation

GNRI was computed using the following formula: GNRI = [1.489 × serum albumin (g/L)] + [41.7 × body weight (kg)/ideal body weight (kg)], where ideal body weight is equal to 22 times the height (in meters) squared (14). If the subject’s present weight is more than their ideal weight, the weight-to-ideal weight ratio is equal to one.

### Assessment of outcome—total testosterone

The Centres for Disease Control and Prevention (CDC) developed an isotope dilution liquid chromatography tandem mass spectrometry (ID-LC-MS/MS) technique to measure the levels of total testosterone in serum for routine examination. This method has been particularly designed for samples with a high rate of flow, and has consistently shown a high level of accuracy and precision over a long period of time. The technique has received certification from the CDC Hormone Standardisation Programme (HoSt) and can be traced back to certified reference material acquired from the Australian National Measurement Institute (ANMI) M914 for testosterone. For a comprehensive examination of quality control and quality assurance in the NHANES laboratory and medical technical personnel manual of procedure (LPM), please refer to the following link: https://wwwn.cdc.gov/nchs/data/nhanes/2013-2014/labmethods/TST_H_MET_TotaL_Estradiol_and_Total_Testosterone. Kindly see the PDF document.

### Definition of other variables

Previous research has identified a number of variables that are taken into account in the model, including the confusion potential variable GNRI and the correlation between serum total testosterone (16–18). Covariates were classified into two categories: sociodemographic and health-related variables. The sociodemographic variables included age, race, marital status, education level, and the poverty income ratio (PIR). The health-related variables encompassed body mass index (BMI), smoking status, drinking status, hypertension, diabetes mellitus, physical activity, sleep duration, triglycerides (TG), high-density lipoprotein cholesterol (HDL-C), and low-density lipoprotein cholesterol (LDL-C).

The participants were categorized by race as non-Hispanic White, non-Hispanic Black, Mexican American, other Hispanic, and other races. Educational level was classified as below high school education, completion of high school education, or above high school education. Smoking status was classified as never, former, or current smoker according to the question “At least 100 cigarettes in your lifetime” and “Are you a current smoker”. Alcohol consumption was determined according to the following questions: “At least 12 alcoholic beverages per year?”, “At least 12 alcoholic drinks in your lifetime?”, and “Frequency of alcohol consumption in the past 12 months”. This was used to classify individuals as never drinking, former drinking, or current drinking. Body mass index (BMI) was divided into three categories: ≤24.9, 25-29.9, and ≥30 kg/m².

A history of hypertension was determined based on the following criteria: a previous diagnosis of hypertension, current medication for hypertension, or a systolic or diastolic blood pressure reading of ≥140/90 mmHg, respectively. Participants who were informed by a physician that they had diabetes, or had a fasting plasma glucose level of ≥126 mg/dL, or used insulin or medication for glycemic control, or had a glycated hemoglobin level of ≥6.5 were considered to have diabetes. Physical activity was categorized as vigorous, moderate, and inactive based on the subject’s performance of any activity that resulted in profuse sweating or a large increase in breathing or heart rate, or that resulted in slight sweating or a moderate increase in heart rate. Sleep duration was categorized into three categories: (1) < 7 hours, (2) 7-9 hours, and (3) > 9 hours (19).

### Statistical analyses

The study reported continuous variables as means with standard deviations and categorical variables as proportions. The entire GNRI dataset was divided into quartiles, with the first quartile (Q1) representing the minimum value. The chi-square test was employed to categorize the GNRI groups for categorical variables, while the t-test was utilized for continuous variables. The basis for comparison was the difference between the four-digit values. We conducted weighted linear regression analysis to assess the association between GNRI and continuous values of TT levels. Three models were utilized in this study to account for various factors. Model 1 did not consider any additional variables. The second model was adjusted for age, race, education level, PIR, and marital status. Model 3 was derived from Model 2 and further modified to include variables such as BMI, HDL-C, LDL-C, TG, diabetes, hypertension, smoking status, drinking status, physical activity, and sleep duration. Regression Model 3 was employed to examine any nonlinear associations between GNRI and testosterone by utilizing limited cubic splines. Additionally, interactions were assessed using log-likelihood ratio tests, stratified by the presence or absence of a prior history of chronic diseases such as hypertension and diabetes, as well as BMI. Ultimately, sensitivity analyses were conducted using unweighted data to verify the reliability of the weighted results. The statistical analyses were performed using the R programme (http://www.R-project.org, The R Foundation) and Free Statistics software version 1.3. Statistical significance was determined by a p-value < 0.05, calculated using a two-tailed test.

## Results

### Demographic and clinical characteristics of study participants

The study comprised a total of 829 people. **Table 1** displays the fundamental features of each category. The weighted sample of 20,631,737 participants across the three survey cycles is representative of the uninstitutionalised US population, with the majority being non-Hispanic white (79.81%). The mean age of the respondents was 68.624 ± 6.221 years, and the mean TT level was 441.400 ± 199.919 ng/dL. The participants in Q4 exhibited the highest TT level, with a mean of 457.460 ± 184.962 ng/dL. Furthermore, they were more likely to be overweight (45.50%). A comparison of the GNRI classification of the four groups based on their TT levels, race, drinking status, and other factors such as diabetes and sleep duration differences yielded statistically significant results (P < 0.05). Subjects with higher levels of GNRI (GNRI Q3 and Q4) exhibited higher triglyceride (TG) and high-density lipoprotein cholesterol (HDL-C) levels, as well as a lower prevalence of diabetes and hypertension.

**Table 1:** Weighted demographic and clinical characteristics in accordance with the GNRI level.

Variables	Overall (n=20631737)	Q1 (n=2730725)	Q2 (n=4851201)	Q3 (n=6064536)	Q4 (n=6985275)	*P*-value
Continuous variable, mean ± SD						
Age (years)	68.624 ± 6.221	69.691 ± 7.097	68.722 ± 6.355	68.684 ± 6.354	68.088 ± 5.588	0.5479
TT (ng/dL)	441.400 ± 199.919	354.280 ± 190.471	440.129 ± 215.289	463.146 ± 198.626	457.460 ± 184.962	<0.0001
ALB (g/L)	42.077 ± 3.111	37.436 ± 2.470	40.721 ± 1.014	42.600 ± 0.642	45.318 ± 1.433	<0.0001
TG (mg/dL)	113.527 ± 61.871	102.940 ± 57.101	108.728 ± 65.190	116.565 ± 57.417	118.361 ± 64.558	0.4034
HDL-C (mg/dL)	52.442 ± 15.573	49.833 ± 16.161	52.549 ± 15.068	52.751 ± 15.278	53.118 ± 15.915	0.4996
LDL-C (mg/dL)	102.611 ± 33.490)	97.385 ± 29.339	100.881 ± 31.721	100.570 ± 31.385	107.626 ± 37.312	0.0824
Categorical variables, %						
Race (%)						0.0005
Mexican American	840012 (4.07)	145045 (5.31)	136678 (2.82)	281915 (4.65)	276375 (3.96)	
Non-Hispanic White	16465176 (79.81)	1823608(66.78)	4035111 (83.18)	4916134 (81.06)	5690323 (81.46)	
Non-Hispanic Black	1620157 (7.85)	439818 (16.11)	440372 (9.08)	398725 (6.57)	341243 (4.89)	
Other Hispanic	664334 (3.22)	87685 (3.21)	161341 (3.33)	132911 (2.19)	282398 (4.04)	
Other Race	1042058 (5.05)	234569 (8.59)	77699 (1.60)	334852 (5.52)	394938 (5.65)	
Education (%)						0.3205
Below high school	3349528 (16.23)	655398 (24.00)	755730 (15.58)	1022397 (16.86)	916003 (13.11)	
Completed high school	4118497 (19.96)	657691 (24.08)	870186 (17.94)	1046589 (17.26)	1544032 (22.10)	
Above high school	13163713 (63.80)	1417637 (51.91)	3225285 (66.48)	3995550 (65.88)	4525241 (64.78)	
PIR (%)						0.0529
≤1.3	2793255 (13.54)	682167 (24.98)	670518 (13.82)	668781 (11.03)	771789 (11.05)	
1.31-3.5	7400676 (35.87)	1056362 (38.68)	1854718 (38.23)	2350483 (38.76)	2139113 (30.62)	
>3.5	10437806 (50.59)	992195 (36.33)	2325965 (47.95)	3045272 (50.21)	4074374 (58.33)	
Marital (%)						0.3479
Married/Live with partner	16735116 (81.11)	2046987 (74.96)	3807452 (78.48)	4994145 (82.35)	5886533 (84.27)	
Widowed/Divorced/Separated	3160298 (15.32)	548627 (20.09)	954852 (19.68)	852756 (14.06)	804063 (11.51)	
Never married	736323 (3.57)	135112 (4.95)	88897 (1.83)	217635 (3.59)	294679 (4.22)	
Smoking (%)						0.1149
Never	7267699 (35.23)	789475 (28.91)	1745475 (35.98)	2098473 (34.60)	2634276 (37.71)	
Former	10852184 (52.60)	1285788 (47.09)	2350747 (48.46)	3470553 (57.23)	3745097 (53.61)	
Now	2511854 (12.17)	655462 (24.00)	754979 (15.56)	495511 (8.17)	605902 (8.67)	
Drinking (%)						0.0362
Never	1188266 (5.76)	284281 (10.41)	276801 (5.71)	275410 (4.54)	351775 (5.04)	
Former	3920304 (19.00)	846456 (31.00)	641097 (13.22)	1141744 (18.83)	1291007 (18.48)	
Now	15523167 (75.24)	1599988 (58.59)	3933303 (81.08)	4647383 (76.63)	5342494 (76.48)	
Diabetes (%)						0.0101
No	14408824 (69.84)	1389950 (50.90)	3607462 (74.36)	4269435 (70.40)	5141978 (73.61)	
Yes	6222913 (30.16)	1340775 (49.10)	1243739 (25.64)	1795101 (29.60)	1843298 (26.39)	
Hypertension (%)						0.1773
No	9260190 (44.88)	893239 (32.71)	2190072 (45.14)	2590383 (42.71)	3586496 (51.34)	
Yes	11371548 (55.12)	1837486 (67.29)	2661129 (54.86)	3474153 (57.29)	3398779 (48.66)	
Physical activity (%)						0.4218
Vigorous	2870896 (13.91)	191553 (7.01)	734398 (15.14)	739727 (12.20)	1205219 (17.25)	
Moderate	6986065 (33.86)	798428 (29.24)	1625205 (33.50)	2129250 (35.11)	2433182 (34.83)	
No	10774776 (52.22)	1740744 (63.75)	2491599 (51.36)	3195559 (52.69)	3346875 (47.91)	
Sleep duration (%)						0.0205
<7hours	4416212 (21.40)	993203 (36.37)	1246744 (25.70)	1026436 (16.93)	1149830 (16.46)	
7-9hours	15273360 (74.03)	1614937 (59.14)	3376860 (69.61)	4704497 (77.57)	5577067 (79.84)	
>9hours	942165 (4.57)	122585 (4.49)	227598 (4.69)	333604 (5.50)	258379 (3.70)	
BMI status (%)						0.4382
≤24.9	4879839 (23.65)	617691 (22.62)	1342298 (27.67)	1242717 (20.49)	1677132 (24.01)	
25-29.9	8231057 (39.90)	915747 (33.53)	1685983 (34.75)	2450991 (40.42)	3178335 (45.50)	
≥30	7520841 (36.45)	1197286 (43.84)	1822920 (37.58)	2370828 (39.09)	2129808 (30.49)	

Values are presented as mean ± standard deviation or n (%).

GNRI, geriatric nutrition risk index; Q, quartile; PIR, poverty to family income of ratio; BMI, body mass index; HDL-C, high-density lipoprotein cholesterol; LDL-C, low-density lipoprotein cholesterol; TG, triglyceride; TT, total testosterone; ALB, albumin.

### The association between geriatric nutrition risk index and total testosterone

**Table 2** illustrates the β coefficient and 95% confidence intervals for the correlation between GNRI and TT in the three regression models. The results demonstrated an independent, positive correlation between GNRI and testosterone across the three adjusted models. In model 1, β was found to be 7.21 with a 95% CI of 3.66 to 10.76, in model 2 β was 6.48 with a 95% CI of 2.74 to 10.23 and in model 3, a significant positive association between GNRI and TT was observed (β = 5.59, 95% CI: 2.16, 9.01; p < 0.05). In models 1, 2, and 3, the β values of the other three groups (Q2, Q3, and Q4) were found to be significantly different from Q1 (all p < 0.05). Furthermore, the two highest GNRI groups (Q3 and Q4) exhibited significantly elevated testosterone levels in comparison to the lowest GNRI group (Q1).

**Table 2:** Multivariate regression analysis of GNRI with testosterone.

Exposure	Model 1 β (95%CI) *P*-value	Model 2 β (95%CI) *P*-value	Model 3 β (95%CI) *P*-value
GNRI	7.21 (3.66-10.76) <0.001	6.48 (2.74-10.23) 0.001	5.59 (2.16-9.01) 0.003
GNRI quartile			
Q1	Ref	Ref	Ref
Q2	85.85 (30.24-141.46) 0.003	78.78 (26.50-131.05) 0.004	70.15 (11.33-128.97) 0.022
Q3	108.87 (55.86-161.87) <0.001	101.06 (47.62-154.49) <0.001	104.40 (53.66-155.13) <0.001
Q4	103.18 (68.95-137.41) <0.001	94.76 (59.78-129.75) <0.001	84.83 (49.63-120.03) <0.001
*P* for trend	<0.001	0.002	0.005

CI, confidence interval; Ref, reference group; GNRI, geriatric nutrition risk index.

Model 1 adjust for: None.

Model 2 adjust for: Age, Race. Marital, PIR, Education.

Model 3 adjust for: Age, Race, Marital, PIR, Education, BMI status, Smoking status, Drinking status, Triglyceride, HDL-C, LDL-C, Hypertension, Diabetes, Sleep Duration, Physical Activity.

### Nonlinear relationship between GNRI and total testosterone in the elderly

**Figure 2:**
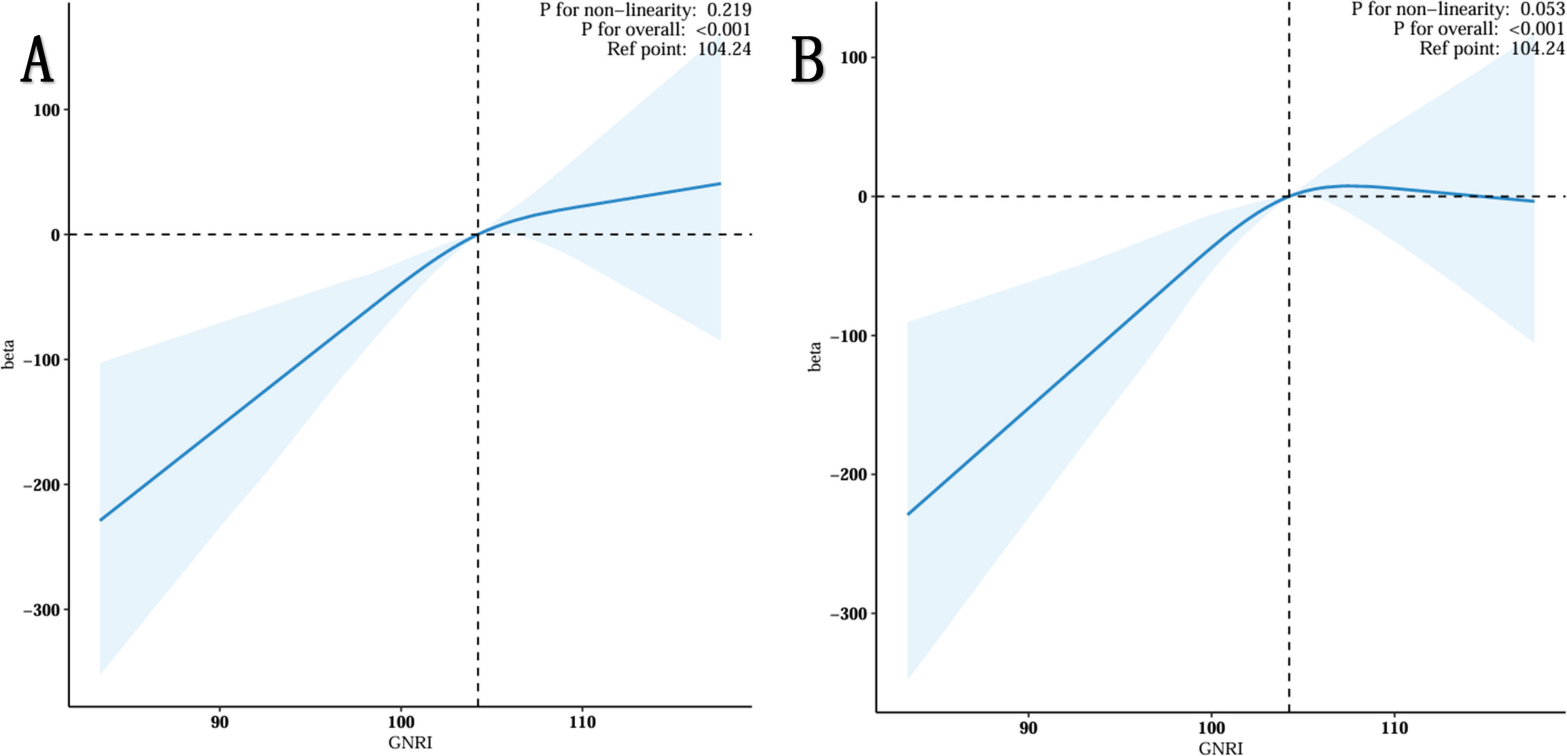
Restricted cubic spline fitting for the association between GNRI with testosterone levels. **(A)** No adjustment. **(B)** Adjust for Age, Race, Marital, PIR, Education, BMI status, Smoking status, Drinking status, Triglyceride, HDL-C, LDL-C, Hypertension, Diabetes, Sleep Time and Physical Activity.

The restricted spline regression model depicted in **Figure 2** demonstrated a nonlinear, positive association between GNRI and total testosterone in the elderly population (p-value for overall: <0.001) without adjustment for any covariates (**Figure 2A**). Once all confounding factors were controlled for (see **Figure 2B**), the positive correlation between GNRI and TT remained significant (p-value <0.001). Furthermore, as GNRI increased, TT growth slowed. This phenomenon occurred when GNRI was greater than 104.24.

### Subgroup analysis

To ascertain the stability of the association between GNRI and TT levels across different BMI statuses, diabetes and hypertension in the elderly, subgroup analysis revealed that no diabetes (p=0.003), hypertension (p < 0.001), BMI ≤ 24.9 (p=0.04), and TT levels (p < 0.001) were significantly associated with GNRI in the elderly. It may be the case that this particular population is particularly vulnerable to GNRI, resulting in elevated TT levels. For example, an additional unit of a BMI of 24.9 or less is associated with a 7.34-fold increase in TT (beta = 7.34, 95% CI: 0.43, 14.25, p = 0.04). Additionally, no significant interaction was observed (p > 0.05). A detailed analysis of the results is presented in **Table 3**.

**Table 3:** Subgroup analysis of GNRI and serum testosterone levels (stratified by diabetes, hypertension, and BMI).

Variables	β (95%CI)	*P* value	*P* for interaction LRT
**Diabetes**			0.246
No	6.85 (2.66, 11.03)	0.003	
Yes	0.78 (-5.79, 7.35)	0.8	
**Hypertension**			
No	2.15 (-4.93, 9.22)	0.53	0.242
Yes	8.64 (4.22, 13.07)	<0.001	
**BMI**			0.683
<=24.9	7.34 (0.43, 14.25)	0.04	
25-29.9	3.63 (-3.34, 10.61)	0.29	
>=30	4.71 (-2.20, 11.62)	0.17	

The model adjusts for all covariables except the stratified variables.

### Sensitivity analyses

As demonstrated in **Table 4**, the GNRI demonstrated a consistent association with TT when unweighted data were analyzed using repeat analysis (beta = 3.78, p = 0.009). Furthermore, in the correct all covariate model, the GNRI remained stable when analyzed using repeat analysis (beta = 3.09, p = 0.031).

**Table 4:** The comparison between weighted and unweighted analysis for detection of sensitivity.

Model	Weight	Total testosterone (ng/dL)
		β (95%CI) *P*-value
Model 1	Weighted	7.21 (3.66-10.76) <0.001
	Unweighted	3.78 (0.95-6.6) 0.009
Model 2	Weighted	5.59 (2.16-9.01) 0.003
	Unweighted	3.09 (0.3-5.85) 0.031

Model 1 adjust for: None.

Model 2 adjust for: Age, Race. Marital, PIR, Education, BMI status, Smoking status, Drinking status, Triglyceride, HDL-C, LDL-C, Hypertension, Diabetes, Sleep Duration and Physical Activity.

## Discussion

This study conducted a retrospective cohort analysis with 829 elderly individuals to evaluate their nutritional condition using the GNRI. An analysis of a representative sample of 206,317 instances of serum testosterone in the older population of the United States produced the subsequent findings. The study originally utilized a weighted logistic regression analysis to investigate the correlation between various dietary scores and the overall levels of testosterone in older people. In addition, the limited cubic spline regression model demonstrated a non-linear relationship between GNRI and TT. Subgroup analysis demonstrated a noteworthy correlation between GNRI and mortality risk in several subgroups of older individuals with hypertension. Sensitivity studies were performed to confirm the strength and reliability of the study’s results.

Malnutrition remains a significantly under-recognized but pivotal issue affecting the elderly population, and its association with disease is garnering mounting research focus (8). The GNRI was initially developed to assess the risk of malnutrition-related comorbidities in elderly individuals and has since evolved into a critical predictor of a variety of disease entities. The nutritional status of elderly individuals can be more comprehensively and reliably assessed when the GNRI is used in conjunction with serum albumin weight and height (20). As far as we know, there have been no epidemiological studies that have shown a connection between GNRI and serum TT levels. However, Tetsuo Hayashi (21) found that decreased serum albumin levels, a variable associated with GNRI, were associated with decreased circulating levels of testosterone. Grossmann (22) pointed out that weight loss can result in total testosterone is proportional to the amount of weight loss of significant increase, especially in the morbidly obese men. The current investigation found a direct and positive relationship between GNRI, which is determined using blood albumin, weight, and height, and serum TT levels.

The precise mechanism by which GNRI and the positive correlation between TT are linked remains unclear. However, it is possible that the following mechanism may be involved. Steroid hormones, sometimes referred to as steroid hormones, are a group of chemicals characterized by a cyclopentane polyhydrophenanthrene structure that is synthesized from cholesterol by the action of cytochrome P450 enzymes. The categories primarily consist of progesterone, oestrogen, androgen, and corticosteroids (23). Once they are released from steroid cells, bioactive steroids are mostly carried in the circulation by albumin, sex hormone-binding globulin (SHBG), and corticosteroid-binding globulin (CBG) (24). Thus, Human Serum Albumin (HSA) plays a crucial role in transporting testosterone and other sex hormones (25). In comparison to other proteins, such as CBG and SHBG, HSA exhibits a relatively low affinity for steroid hormones, with affinities that are three or four orders of magnitude lower. However, as one of the most abundant proteins in the blood, albumin accounts for approximately 60% of the protein in the loop, with a plasma concentration that is approximately 1000 times that of the other major steroid binding protein. (26). Therefore, it is expected that disorders such as severe malnutrition, cirrhosis, nephrotic syndrome, and other critical illnesses, which lead to reduced levels of plasma albumin, would change the way plasma testosterone is distributed in these individuals (27). Manni’s research determined that albumin-bound sex steroids are accessible for absorption by the majority of tissues, but SHBG-bound sex steroids are not. Thus, it was shown that the concentrations of serum albumin had a significant impact on the maintenance of serum bioactive sex steroid levels (28). Hayashi and Yamada (21) demonstrated that albumin levels were a more significant factor than SHBG levels in influencing bioavailable sex steroid levels in men in their 60s and 70s. I In the present study, higher serum albumin levels were accompanied by higher GNRI scores, which may reflect a positive correlation between GNRI and TT levels. However, more empirical investigations are necessary to validate the precise mechanism that connects GNRI and TT.

In addition, stratified analyses were conducted to evaluate other potential confounding variables that may have influenced the outcomes. The present study demonstrated that the correlation between GNRI and TT was more pronounced in non-diabetic subjects than in diabetic subjects. This may be attributed to the effect of diabetic hypoglycaemic drugs on blood sugar levels, which in turn affects the GNRI index (29). The aforesaid study also revealed that the association of GNRI with TT was higher in the non-hypertensive population. The Geriatric Nutritional Risk Index (GNRI) is considered a very reliable method for evaluating the nutritional status of older people with chronic illnesses. It is particularly associated with the malnutrition-inflammation score (30). It was found that levels of inflammatory factors, including IL-6 and TNF-α, were significantly upregulated in hypertensive patients (11), and these inflammatory factors were in turn associated with decreased testosterone levels (31). Consequently, it can be postulated that inflammatory factors may be implicated in the association between GNRI and TT levels in hypertensive patients. Furthermore, this research demonstrated that the correlation between GNRI and TT was particularly noteworthy among those with a BMI of 24.9 or below. A low BMI may indicate malnutrition, which may be caused by the loss of muscle and adipose tissue (32). Multiple cross-sectional investigations conducted on middle-aged and older men have consistently shown a negative correlation between body mass index (BMI) and the levels of total testosterone (33–35), and at the same time, lower BMI populations are usually accompanied by lower albumin levels. (36). Among the various indicators used to assess nutritional status, serum albumin level and BMI are typically regarded as the most important (37), and GNRI can well integrate BMI and serum albumin level to assess a person’s nutritional status. Therefore, for the elderly population with lower BMI, their nutritional status may be worse, and their GNRI score and TT level may be more closely related. Additional research is necessary to examine the correlation between GNRI and TT levels in persons with different BMI.

By establishing a novel association between GNRI and TT levels in an elderly male population in the United States, this study makes a substantial contribution to the field. In this population, GNRI has the potential to serve as a TT indicator. Additionally, the GNRI provides a thorough evaluation of the nutritional risk associated with the geriatric. Nevertheless, additional research is necessary to determine its precise function in the measurement of TT levels and the replacement of existing methods. In order to evaluate the diagnostic accuracy and clinical utility of GNRI in comparison to other measures, additional longitudinal and comparative studies are necessary. The GNRI can be used as a supplementary diagnostic instrument to provide a patient with better understanding of their nutritional status. The inclusion of GNRI in the diagnostic procedure may potentially aid in the identification of individuals who may benefit from further evaluation or are at higher risk, given the well-established association between steroid hormones and nutritional status.

There are several benefits to this study. First, a comprehensive, nationally representative database acquired using defined techniques yields more reliable and persuasive results. Secondly, the GNRI, which is an indication of malnutrition in older persons rather than a single sign, was used to define malnutrition. Third, using stratified analysis, we assessed the relationship between GNRI and TT levels in several demographic subgroups after completely accounting for confounding variables. Lastly, a comparison of the weighted and unweighted data verified the stability of the findings.

There are several further restrictions on our investigation. First of all, because this was cross-sectional research, we were unable to prove causation. Second, because it is based on the NHANES database, the poll is only available to those living in the United States. Third, even though we adjusted for a number of factors, bias may still arise from other unmeasured confounders. The findings of this study will require confirmation by prospective investigations in the future.

## Conclusion

This cross-sectional study demonstrates that GNRI is positively associated with total testosterone levels in US adults older than 60 years of age. The results make up the previous research, but these studies still need a larger prospective cohort for validation.

## Data Availability

The original contributions presented in the study are included in the article/**Supplementary Material**. Further inquiries can be directed to the corresponding author.
